# Ion Superhighways in a Hierarchical Polymer‐Ceramic Membrane Enable Rapid and Selective Lithium Extraction

**DOI:** 10.1002/advs.76649

**Published:** 2026-07-20

**Authors:** Xinxin Wei, Jiawei Sun, Min Wei Boey, Xiaolu Li, Minji Kim, Junghwan Kim, Hongjiang Chen, Patrick H.‐L. Sit, Yixiang Wang, Zhigang Li, Ze‐Xian Low, Jason Chun‐Ho Lam, Alicia Kyoungjin An

**Affiliations:** ^1^ School of Energy and Environment City University of Hong Kong Kowloon Hong Kong China; ^2^ Department of Chemical and Biological Engineering The Hong Kong University of Science and Technology Hong Kong China; ^3^ Department of Chemical and Biomolecular Engineering Yonsei University Seoul Republic of Korea; ^4^ City University of Hong Kong Shenzhen Research Institute Shenzhen China; ^5^ Department of Mechanical and Aerospace Engineering The Hong Kong University of Science and Technology Hong Kong China; ^6^ State Key Laboratory of Materials‐Oriented Chemical Engineering National Engineering Research Center for Special Separation Membrane Nanjing Tech University Nanjing China

**Keywords:** brine, ceramic, electrodialysis, DLE, lithium, materials science, membrane, selectivity, taxonomy

## Abstract

Lithium recovery from complex brines is a key challenge for the clean energy transition. Conventional polymer membranes lack selectivity, while highly selective ceramic solid electrolytes are brittle and difficult to scale. Here we address this gap by creating a flexible polymer‐ceramic membrane that embeds *Li*_1.3_ *Al*_0.3_ *Ti*_1.7_(*PO*_4_)_3_ (LATP) ceramic particles into a processable PDMS polymer matrix. This hierarchical transport architecture creates an ion ‘superhighway’, providing selective Li^+^ conduction through LATP while the PDMS phase imposes an additional interfacial penalty on competing ions. In lithium‐selective electrodialysis, the membrane achieves a Li^+^ flux of 968 mmol m^−^^2^ h^−^^1^ and an exceptional Li^+^/Mg^2+^ selectivity of 2832 and remains stable for 480 h of continuous lithium extraction from real seawater brine. This strategy translates the selectivity of solid electrolytes into a scalable membrane, enabling efficient direct lithium extraction.

## Graphical abstract

A flexible PDMS‐LATP polymer‐ceramic membrane creates lithium‐ion “superhighways” for rapid and selective extraction from complex brines. The amorphous PDMS matrix suppresses nonselective leakage and stabilizes the ceramic network, while LATP provides preferential Li+ conduction. This hierarchical architecture enables high‐flux electrodialysis, exceptional Li^+^/Mg^2+^ selectivity, and long‐term operation in real seawater brine.

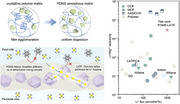

## 1 Introduction

*Lithium*, a white gold, is a critical enabling material for electrified transportation and grid‐scale energy storage, and demand projections have intensified interest in expanding lithium supply beyond conventional hard‑rock mining and high‑grade continental brines [1, 2, 3]. A substantial fraction of global lithium resources, however, resides in low‐grade and compositionally complex aqueous streams, including salt‐lake brines, geothermal fluids, industrial brines, and seawater‐derived concentrates. In these feedstocks, lithium is typically present at low concentrations and accompanied by overwhelming excesses of competing ions such as Na^+^, Mg^2+^, K^+^, and Ca^2+^, making selective recovery intrinsically challenging. Conventional solar evaporation remains widely used for certain high‐quality brines, but it is slow, land‐intensive, and sensitive to climate; moreover, it becomes inefficient or impractical when brines are dilute, highly variable, or dominated by competing ions [4]. These constraints have motivated the development of **direct lithium extraction (DLE)** technologies that can operate on low‐grade or complex brines with improved selectivity and footprint [5].

Among emerging DLE approaches, electrically driven processes are attractive because they can offer controllable ion transport through applied potential and can be modularly integrated with existing desalination and brine‑management infrastructure. In particular, electrodialysis (ED) uses ion‐selective membranes to drive ionic flux under electric fields and has been explored for lithium enrichment and ion separation [6, 7, 8]. However, its broader implementation remains limited by a persistent membrane challenge: lithium must be transported selectively at practically relevant fluxes in the presence of large excesses of chemically similar competing cations and under the high ionic strength conditions characteristic of real brines. Conventional polymeric ion‐exchange membranes often struggle to satisfy these requirements simultaneously, because improvements in permselectivity are frequently accompanied by losses in conductivity or permeability, while swelling, defect‐mediated leakage, and concentration polarization further degrade performance under realistic operating conditions. The central challenge is not merely to create a membrane that conducts lithium, but to ensure that lithium transport remains selective, robust, and processable in complex aqueous environments [9, 10].

An emerging strategy to break this trade‐off is to exploit inorganic solid‐state electrolytes (ISSE) that exhibit intrinsically high Li^+^ conductivity and strong preference for Li^+^ transport through crystallographic pathways. NASICON *(Na Superionic Conductor)*‐type ceramics such as lithium aluminum titanium phosphate (LATP) *Li*_1.3_ *Al*_0.3_ *Ti*_1.7_(*PO*_4_)_3_ are well established as lithium‐ion conductors in solid‐state batteries and provide percolating frameworks that facilitate Li^+^ migration while excluding many non‐lithium ions by lattice chemistry and site energetics [11, 12, 13, 14, 15, 16]. Translating this “lithium‐conducting solid electrolyte” into aqueous membrane separations could, in principle, enable exceptional Li selectivity. In practice, however, **ceramic electrolyte membranes** face multiple constraints for water applications: (i) brittleness and defect sensitivity that complicate fabrication of thin, large‐area membranes; (ii) interfacial and grain‐boundary defects that create nonselective leakage paths; (iii) chemical degradation or surface reactions in aqueous electrolytes depending on brine composition and pH [17]; and (iv) limited scalability and high cost of fully dense ceramic processing [18, 19]. Overcoming these limitations requires a membrane architecture that preserves the Li^+^‐conducting function of the solid electrolyte while providing mechanical compliance, defect‐free, and scalable manufacturing [20].

A promising route forward is to construct flexible polymer‐ceramic membrane architectures in which lithium‐conducting ceramic domains remain transport‐dominant, while the surrounding polymer phase suppresses defect‐mediated leakage and stabilizes the interface in aqueous media. In this context, the polymer should not be viewed merely as a passive binder. Rather, it governs ceramic dispersion, interfacial continuity, defect formation, and the local solvation environment experienced by ions before they enter the ceramic phase. These considerations are especially important for lithium‐selective transport, where subtle differences in dehydration energetics, charge density, and interfacial partitioning can strongly affect competition between Li^+^ and other cations. An amorphous, electrically insulating, and hydrophobic polymer matrix is particularly attractive in this regard because it can facilitate homogeneous incorporation of ceramic particles without generating crystalline polymer‐rich bypass domains, while also modifying the local dielectric environment at the polymer‐ceramic boundary [21, 22]. Such membrane architecture could enable the membrane‐scale expression of solid‐electrolyte ion sieving in a processable and mechanically compliant format.

Here, we present a flexible polymer‐ceramic membrane architecture that translates the intrinsic lithium selectivity of LATP into a processable electrodialysis membrane for complex brines. By dispersing surface‐modified LATP particles within an amorphous PDMS matrix, we sought to preserve the Li^+^‐conducting function of the NASICON lattice while suppressing defect‐mediated leakage, improving aqueous stability, and introducing mechanical compliance at membrane scale. We show that this composite architecture enables hierarchical ion selection, in which the PDMS phase regulates interfacial ion entry and the LATP phase governs selective Li^+^ transport through its crystallographic conduction network. Using electrodialysis experiments in model salt solutions and authentic seawater reverse‐osmosis brine, together with electrochemical and thermodynamic analyses, we demonstrate that the membrane couples solid‐state lithium selectivity with polymer processability in a format suitable for scalable lithium recovery from complex aqueous feed streams.

## 2 Results

### 2.1 An Amorphous Polymer Matrix Enables Defect‐Suppressed LATP Membranes

Translating the intrinsic lithium selectivity of LATP into an aqueous electrodialysis membrane requires more than incorporating a lithium‐conducting ceramic into a polymer film [23]. The membrane must simultaneously preserve continuous LATP transport pathways, suppress non‐selective leakage through interfacial defects, and remain mechanically compliant enough for scalable processing and operation. These requirements are difficult to satisfy with dense LATP ceramics alone, which are brittle, defect‐sensitive, and typically require high‐temperature sintering in mold‐limited formats. We therefore sought a hybrid membrane architecture in which the ceramic phase provides selective Li^+^ conduction, while the surrounding polymer phase maintains interfacial continuity and structural integrity at membrane scale. We selected PDMS as the host matrix because its amorphous and elastomeric character is more compatible with uniform LATP dispersion and defect suppression than semi‐crystalline polymer matrices such as PVDF‐HFP or PAN [24, 25].

**FIGURE 1:**
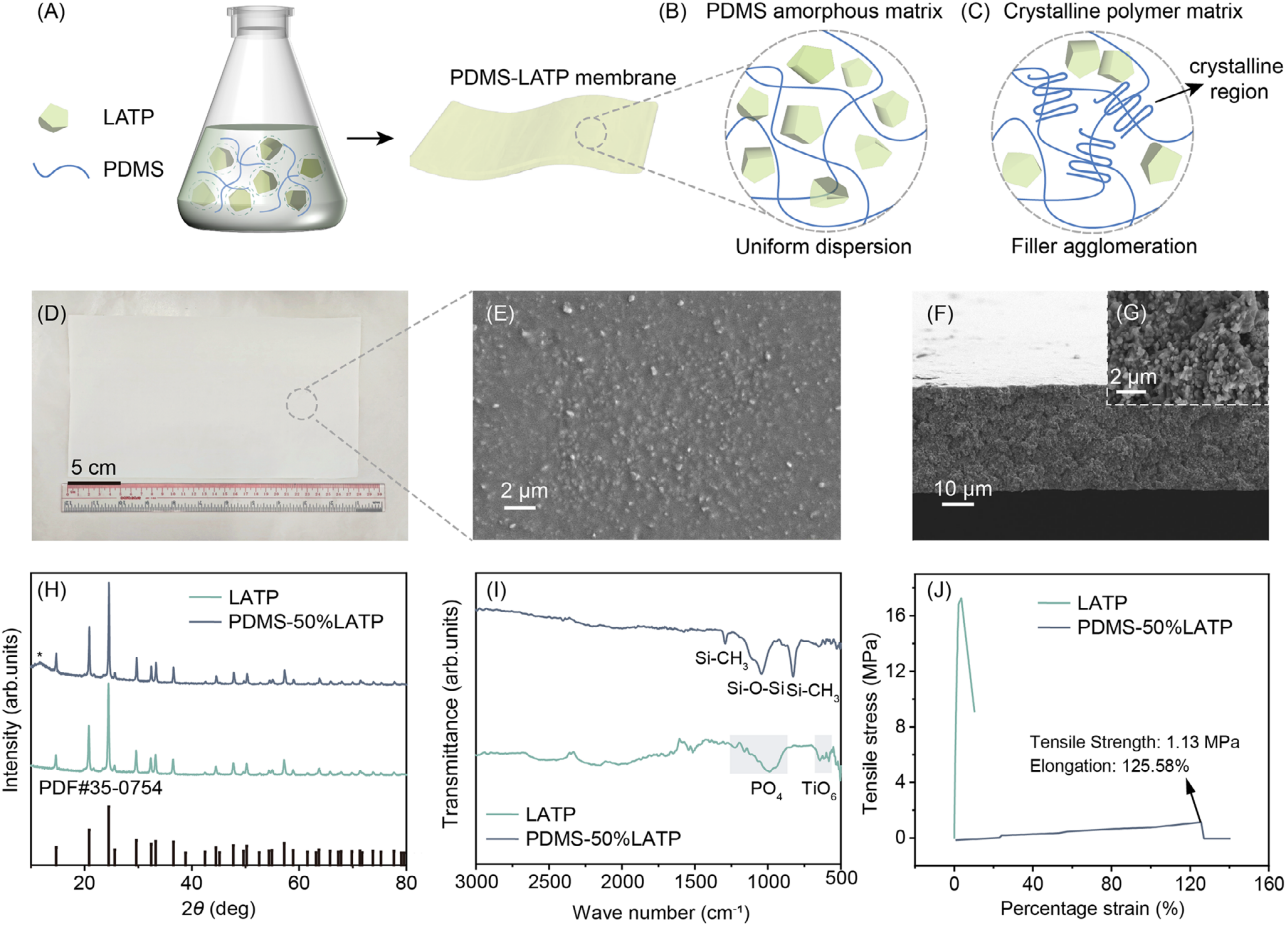
Characterization of LATP ceramic membrane and PDMS‐50%LATP membrane. (A) Schematic of the PDMS‐LATP membrane fabrication process; Comparison of the inherent advantages of (B) amorphous PDMS versus (C) crystalline polymers; (D) Photograph of the freestanding PDMS‐50%LATP membrane; SEM images showing (E) its surface morphology and (F) cross‐sectional view with (G) a magnified region; (H) XRD patterns and (I) FTIR spectra of the membranes; (J) Mechanical strength measured under tensile testing.

To address the challenge of dispersing hydrophilic LATP ceramic particles uniformly in the hydrophobic PDMS matrix, TEOS was employed as a coupling agent to form a silica/siloxane interfacial layer on the LATP surface. The importance of this modification is demonstrated by a direct comparison to an unmodified membrane (see Figure S1 for characterization). Without TEOS modification, the membrane fails structurally. SEM imaging reveals severe LATP agglomeration and surface defects (Figure S1C). This poor integration results in macroscopic phase separation, confirmed by asymmetric surface wetting: one side of the membrane is LATP‐rich and more hydrophilic (contact angle 91°), while the other is PDMS‐rich and highly hydrophobic (108°, Figure S1B). In contrast, the TEOS‐modified membrane is structurally uniform and exhibits consistent hydrophobicity, confirming successful integration. This improved structural integrity is the basis for the membrane's selective transport performance, as the unmodified membrane suffers from catastrophic leakage of competing ions (Figure S15). A mixture of TEOS and LATP at a weight ratio of 1:10 was first dispersed in n‐heptane, which significantly enhanced the viscosity and stability of the LATP particles. This pre‐treated LATP suspension was then blended with a PDMS base/crosslinker mixture (10:1 w/w) to form a homogeneous viscous casting solution. The resulting solution was cast onto a glass plate using a doctor blade and thermally cured at 90°C for 3 h, yielding freestanding membranes with tunable LATP loadings (Figure 1A–D). As LATP loading increased from 10 to 70 wt.%, SEM images revealed a progressive increase in surface protrusions formed by LATP particles (Figure S2). At intermediate loading (50 wt.% LATP), cross‐sectional EDS mapping revealed a continuous distribution of phosphorus (P) signals from LATP spanning the entire membrane thickness, embedded within a uniform silicon (Si) signal from the PDMS matrix (Figure S3). This confirms the formation of an interconnected ceramic network, providing the structural basis for through‐plane ion conduction. Furthermore, high‐magnification SEM images show that these LATP particles are evenly distributed within the PDMS matrix, with no obvious voids or interfacial defects (Figure 1E–G). This homogeneous architecture is critical, as selective transport requires both a percolated LATP network for Li^+^ conduction and a defect‐suppressed polymer matrix to prevent non‐selective bypass. In contrast, increasing the LATP content to 70 wt.% led to visible agglomeration, void formation, and loss of mechanical integrity during membrane handling (Figure S2C,D), indicating that excessive ceramic loading compromises the polymer's ability to maintain a coherent transport architecture. While these structural features are consistent with the deterioration of selective transport pathways, we do not directly resolve defect‐specific leakage in the present study.

Structural characterization further confirmed that membrane fabrication preserved the LATP transport phase. XRD patterns of PDMS‐50%LATP retained the characteristic reflections of rhombohedral LATP, while the broad hump centered near 11° reflected the amorphous PDMS matrix (Figure 1H) [26]. No additional diffraction peaks were detected, indicating that composite formation did not alter the crystalline LATP framework [27]. FTIR spectra likewise showed characteristic LATP‐associated Ti‐O and P‐O features in the composite membrane, consistent with chemical incorporation of the ceramic phase without loss of its structural identity (Figure 1I) [28]. These results indicate that the membrane‐processing route preserves the selective ceramic conductor while embedding it in an amorphous matrix capable of minimizing non‐selective bypass pathways.

The role of the polymer phase became clearer from comparison with semi‐crystalline polymer matrices. LATP‐based membranes prepared using PVDF‐HFP or PAN showed filler agglomeration, phase separation, and microcracking (Figure 1C and Figures S4 and S5), consistent with poor accommodation of high ceramic loadings during membrane formation. By contrast, PDMS‐LATP membranes remained flexible and mechanically robust, unlike both brittle dense LATP ceramics (Figure S6) and the fragile semi‐crystalline polymer composites. Tensile testing confirmed the low‐modulus, deformable character of the PDMS‐based membrane (Figure 1J), which is advantageous for pressure‐free electrodialysis operation and stack sealing. These comparisons show that the polymer phase does not merely act as a passive binder; rather, the amorphous PDMS matrix stabilizes a mechanically compliant and defect‐suppressed LATP network that is necessary for membrane‐scale expression of lithium‐selective transport.

### 2.2 Selective Lithium Transport Emerges From a Flexible Hybrid Conduction Network

**FIGURE 2:**
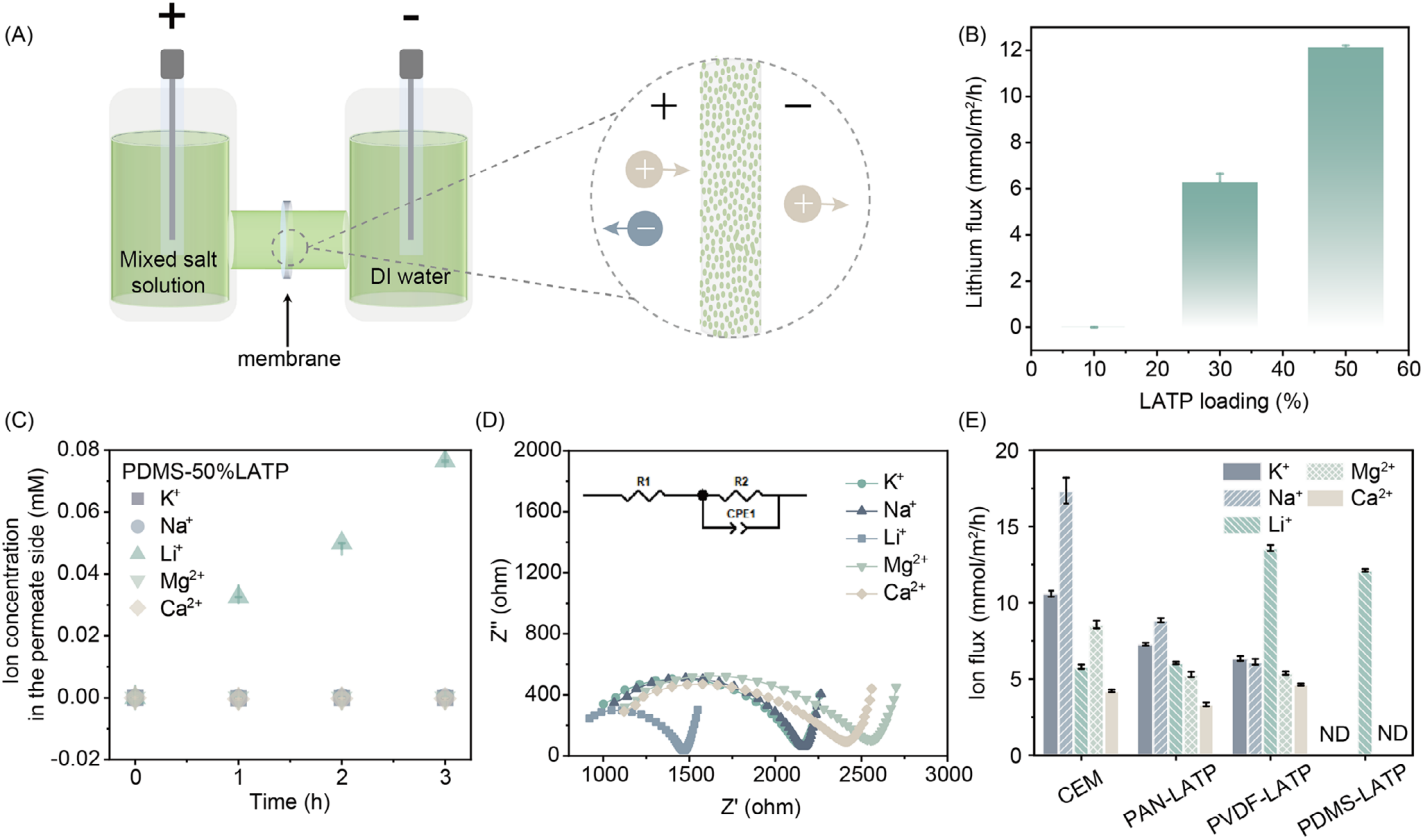
H‐cell evaluation of PDMS‐LATP membrane performance for selective lithium extraction. (A) Schematic of the H‐cell experimental setup: The membrane was mounted between two chambers (feed side: 0.5 M mixed salt solution containing 0.1 M each of LiCl, NaCl, KCl, MgCl_2_, and CaCl_2_; receiving side: DI water) with Pt electrodes applying a 2V electric field; (B) Comparison of lithium flux across membranes with varying LATP filler loadings; (C) Time‐dependent ion concentration profiles in the permeate side using PDMS‐50% LATP membrane; (D) Electrochemical impedance spectroscopy of PDMS‐50%LATP membrane in 0.1 M solutions of KCl, NaCl, LiCl, MgCl_2_, and CaCl_2_; (E) Ion flux of cations for commercial CEM, PAN‐LATP, PVDF‐LATP and PDMS‐LATP membranes.

Selective ion transport under electrodialysis‐relevant conditions depends on whether the defect‐suppressed PDMS‐LATP architecture can preserve ceramic conduction while suppressing non‐selective leakage. Membranes with different LATP loadings were therefore evaluated in a custom H‐cell under an applied potential of 2 V, using a mixed‐salt feed containing LiCl, NaCl, KCl, MgCl_2_, and CaCl_2_ (0.1 M each) and deionized water as the receiving phase (Figure 2A). This configuration was designed to test whether membrane‐scale lithium selectivity could be sustained in the presence of both monovalent and divalent competing cations.

Varying the LATP loading revealed that selective transport depends on a structural balance rather than simply on increasing ceramic content. Pristine PDMS showed negligible cation transport because its dense hydrophobic matrix does not provide continuous ionic pathways [29]. At low LATP loading (10 wt.%), Li^+^ flux remained minimal, indicating that isolated LATP domains were insufficient to establish a percolating conduction network across the membrane (Figure 2B). Increasing the ceramic fraction progressively increased lithium flux, consistent with the formation of connected LATP‐mediated transport pathways. However, this trend did not continue indefinitely: at 70 wt.% LATP, the membrane lost flexibility and became increasingly susceptible to defect formation, which promoted non‐selective ion migration. The highest lithium selectivity was observed at 50 wt.% LATP, where ceramic percolation enabled efficient Li^+^ conduction while the polymer phase still preserved membrane integrity and suppressed bypass transport, indicating that selective lithium transport emerges only when these two conditions are simultaneously maintained (Figure 2C). To distinguish electric‐field‐driven selective transport from passive diffusion, we also examined ion permeation through PDMS‐50%LATP under concentration‐gradient‐driven conditions in the absence of an applied voltage. Over a 4 h test period, any cation transport remained below the quantification limit of our ion chromatography analysis, indicating that the membrane does not function as a simple diffusion pathway for hydrated ions. Therefore, selective extraction requires field‐driven activation of the hybrid conduction network, consistent with the intended electrodialysis mechanism.

As a benchmark, a dense LATP ceramic membrane was tested under identical H‐cell conditions (Figure S7). It exhibited extremely high Li^+^ selectivity, with competing cation fluxes below the analytical detection limit, but at the cost of a significantly lower overall Li^+^ flux compared to the composite membrane. Through‐plane EIS further showed that PDMS‐50%LATP presented the lowest transport resistance to Li^+^ among the tested cations (Figure 2D), confirming that the membrane retained a strong intrinsic preference for lithium conduction after incorporation of LATP into the polymer matrix. The resulting conductivity sequence, Li^+^ > K^+^ > Na^+^ > Mg^2+^ > Ca^2+^, is consistent with the combined influence of ionic hydration, dehydration penalty, and access to LATP‐mediated transport pathways. Together, these results support a mechanism in which electric‐field‐driven Li^+^ migration through LATP is selectively expressed because competing ions experience a greater energetic penalty for interfacial entry and transport.

The importance of the polymer phase choice was further clarified by comparison with commercial and semi‐crystalline polymer‐based membranes [30, 31]. Commercial cation‐exchange membranes and PAN‐LATP composites showed negligible lithium selectivity, while PVDF‐HFP‐LATP provided only limited improvement in Li^+^ flux without effectively suppressing the transport of competing ions (Figure 2E). In contrast, the PDMS‐LATP membrane uniquely combined high lithium permeation with strong rejection of other cations. This comparison shows that adding LATP alone is insufficient to achieve selective lithium transport. Rather, membrane‐scale selectivity emerged only when the ceramic phase was embedded in an amorphous matrix capable of maintaining homogeneous filler dispersion and minimizing non‐selective leakage pathways. Combined with the structural observations in Figure 1, these transport results suggest that the amorphous PDMS matrix is essential for expressing LATP‐mediated selectivity at membrane scale by maintaining filler continuity and suppressing non‐selective leakage.

### 2.3 Hierarchical Interfacial and Lattice Selectivity Governs Li^+^ Transport in the PDMS‐LATP Membrane

**FIGURE 3:**
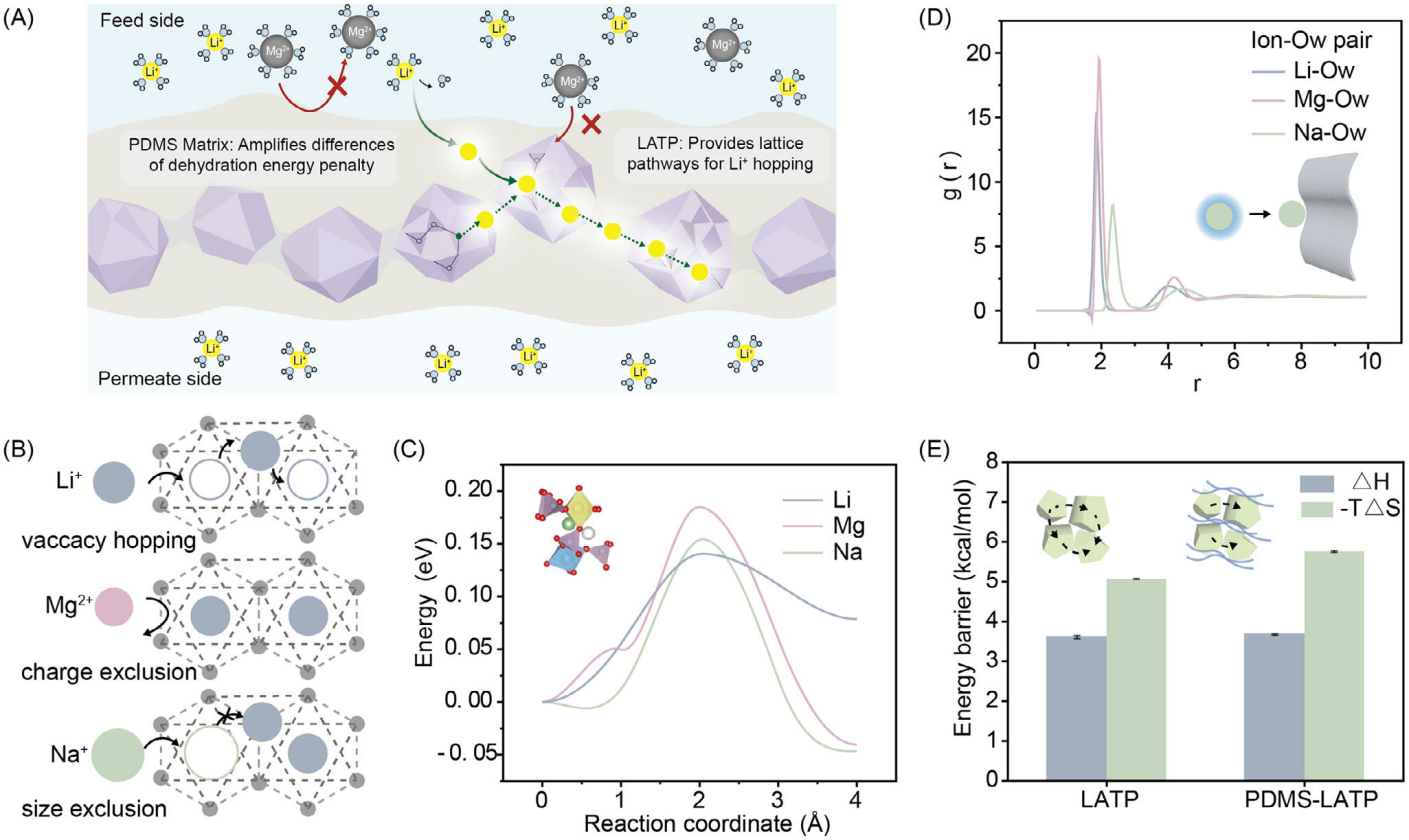
Lithium‐ion transport and selectivity mechanisms in the PDMS‐LATP membrane. (A) Schematic of Li^+^ migration in PDMS‐LATP membrane: dehydration at the surface followed by naked Li^+^ diffusion in LATP channels; (B) Schematic of cation sieving mechanism in LATP; (C) DFT‐calculated diffusion energy barriers for cations in LATP; (D) Cation‐water oxygen radial distribution functions in chloride salt solutions at 298.15 K and 1 bar; (E) Enthalpic and entropic contributions to the free energy barrier for Li^+^ transport in LATP and PDMS‐LATP membranes.

The lithium selectivity of the PDMS‐LATP membrane arises from a hierarchical transport mechanism in which the LATP ceramic lattice serves as the decisive Li^+^‐conducting selective phase, while the polymer interface imposes an additional interfacial barrier to competing ions (Figure 3A). The dominant selective element is the LATP phase, whose rigid NASICON framework contains narrow transport bottlenecks that favor Li^+^ migration while penalizing larger or more highly charged cations [32]. Unlike conventional ion‐exchange membranes, transport through LATP requires ion dehydration before entry into the solid‐state conduction pathway. Under these conditions, selectivity is not governed solely by ionic size: although dehydrated Li^+^ and Mg^2+^ have comparable radii, the higher charge density of Mg^2+^ produces substantially stronger electrostatic exclusion within the LATP lattice (Figure 3B). DFT calculations of migration barriers within LATP supported this picture, yielding the trend Li^+^ < Na^+^ < Mg^2+^ (Figure 3C and Text S9), in agreement with both the transport measurements and previous reports. The calculations were performed using a representative Al‐substituted LATP structural model. These results indicate that selective transport within LATP is governed by coupled size‐sieving and charge‐repulsion effects, with charge discrimination becoming particularly important for multivalent ions. The established trend Li^+^ < Na^+^ < Mg^2+^ aligns well with our experimental observations and existing literature [23], confirming the high selectivity for Li^+^ governed by this coupled effect of size sieving and dominant charge repulsion.

The PDMS phase provides a second, interfacial level of selection before ions access LATP transport channels [22]. Molecular dynamics simulations showed that Li^+^, owing to its lower hydration energy and more labile hydration shell, can more readily undergo hydration‐shell rearrangement at the hydrophobic PDMS interface (Figure 3D). By contrast, Mg^2+^ retains a more strongly bound hydration structure because of its higher charge density, making interfacial entry energetically less favorable. The PDMS matrix therefore does not act as an ion‐conducting phase itself, but rather as a selective interfacial environment that preferentially hinders the approach of strongly hydrated competing ions to the LATP surface. In this way, PDMS amplifies the intrinsic selectivity of LATP by introducing an additional kinetic penalty for ions that are less able to reorganize or shed their hydration shells. This interpretation is further supported by temperature‐dependent transport measurements. To experimentally probe the energetic barriers governing ion transport, we performed Arrhenius analysis for each cation (Figure S8). Specifically, Li^+^ exhibits the lowest activation energy, consistent with its labile hydration shell. In contrast, the divalent cations are much more strongly hydrated and display substantially higher transport barriers. This confirms that the composite membrane architecture imposes a significant energetic penalty on strongly hydrated ions, consistent with the proposed role of the hydrophobic PDMS phase as a selective interfacial gate that amplifies the intrinsic selectivity of the LATP conduction channels.

To further examine how incorporation of PDMS influences Li^+^ transport, transition state theory (TST) was used to compare the free‐energy barriers for lithium migration in dense LATP and PDMS‐LATP membranes based on temperature‐dependent conductivity measurements (Figure S9 and Text S6). For the composite PDMS‐LATP membrane, the extracted enthalpic and entropic terms should be interpreted as apparent transport parameters of the heterogeneous membrane, reflecting the combined effects of LATP‐mediated ion migration, interfacial transfer, particle connectivity, and transport tortuosity, rather than as intrinsic descriptors of a single microscopic hopping event. The enthalpic contribution for PDMS‐LATP remained close to that of pure LATP (Figure 3E), indicating that incorporation of PDMS does not substantially alter the intrinsic Li^+^ migration mechanism within the NASICON lattice. A slight increase in enthalpy is consistent with a modest additional barrier associated with interfacial dehydration or transfer from solution into the ceramic phase. By contrast, the entropy change became more negative in the composite membrane, indicating a greater degree of ordering as Li^+^ moves from aqueous solution into the confined transport environment of PDMS‐LATP. This enhanced ordering likely reflects interfacial confinement imposed by the polymer phase and the more restricted geometry of available conduction pathways in the composite [33]. These thermodynamic results show that PDMS modifies the entry environment and transport organization of Li^+^ without disrupting the fundamental lithium‐conduction character of LATP.

The available structural, electrochemical, and transport results indicate that the LATP phase is the decisive determinant of lithium transport in the PDMS‐LATP membrane, since selective Li^+^ permeation emerges only when the ceramic loading is sufficient to form a percolating network of LATP particles, creating the ‘ion superhighways’ that provide a continuous conduction pathway across the membrane, while low loading leaves the LATP domains too isolated to sustain effective transport. The strong increase in Li^+^ flux at intermediate LATP content, the lowest Li^+^ transport resistance observed by EIS, and the absence of measurable ion permeation under concentration‐gradient‐driven conditions together indicate that lithium transport proceeds primarily through an electrically activated LATP‐mediated network rather than by passive diffusion through the polymer matrix. Within this network, Li^+^ transport is governed by interfacial entry into LATP followed by migration through its crystallographic conduction channels, consistent with the experimentally observed selectivity and the DFT‐calculated barrier trend. The PDMS phase, although not an ion‐conducting phase itself, is essential for enabling this mechanism at membrane scale, because it promotes homogeneous LATP dispersion, suppresses defect‐mediated leakage, and modifies the interfacial ion‐entry environment, as supported by the structural characterization and computational analysis. The lithium selectivity of the composite membrane is therefore best understood as a hierarchical transport process in which LATP provides the decisive Li^+^‐conducting selective phase, while PDMS serves as a structural and interfacial regulator that enables selective transport to be expressed in a robust composite architecture.

### 2.4 Selective Lithium Extraction in a Customized Electrodialysis Stack

**FIGURE 4:**
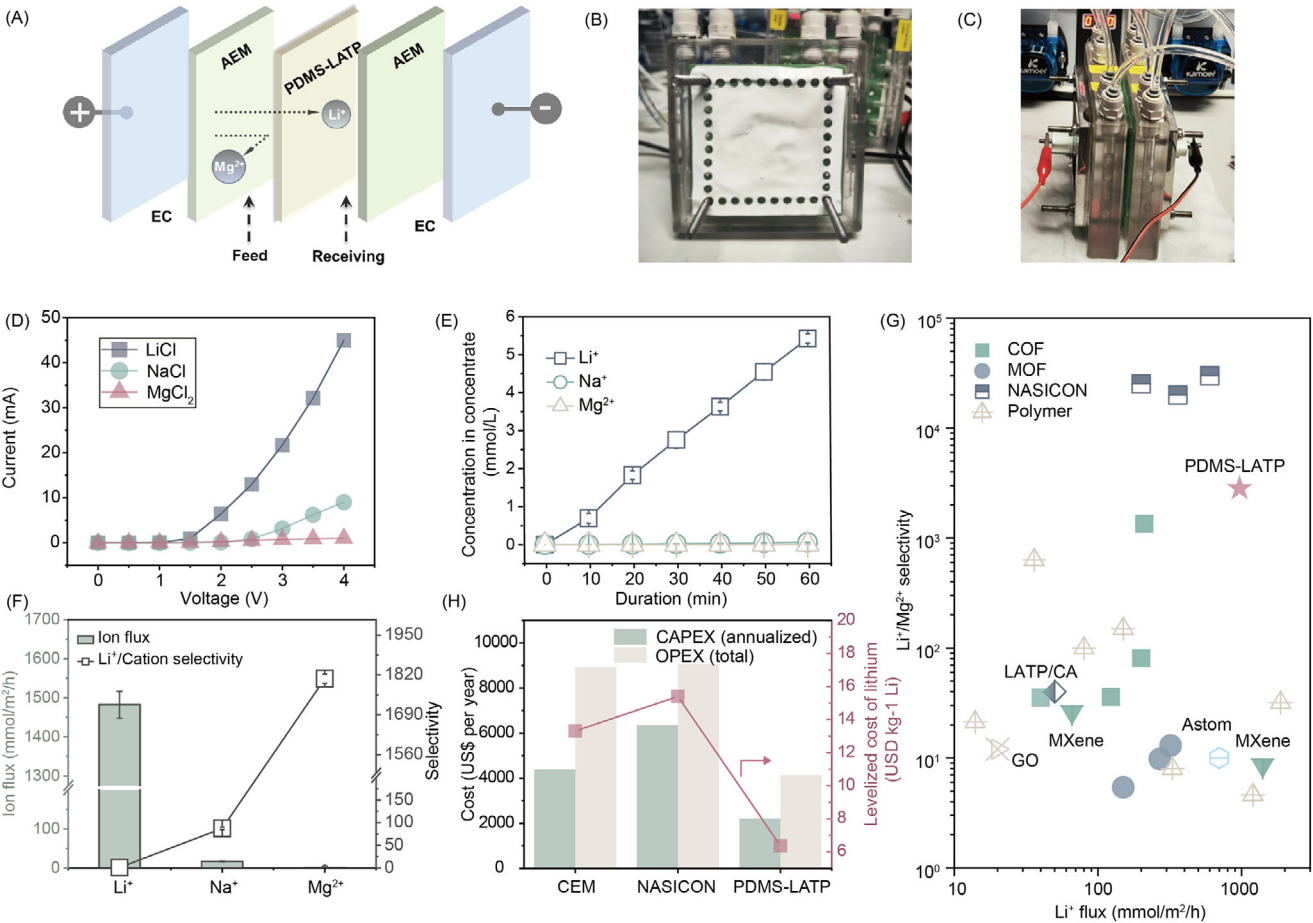
Performance evaluation of the LATP‐PDMS membranes in an electrodialysis stack for selective lithium extraction. (A) Schematic illustration of the single‐pair electrodialysis stack configuration; (B, C) Photograph of the 10 cm × 10 cm LATP‐PDMS membrane integrated in the electrodialysis stack; (D) Curve of current changing with voltage of electrodialysis device for single‐salt solutions (0.1 M LiCl, NaCl, and MgCl_2_). (E) Temporal evolution of cation concentrations in the concentrate compartment for single‐salt solutions (0.1 M LiCl, NaCl, and MgCl_2_); (F) Comparison of ion fluxes and Li^+^/M^n+^ selectivity coefficients in single salt solution; (G) Benchmarking of Li^+^ flux and Li^+^/Mg^2+^ selectivity (pink star) against state‐of‐the‐art membranes from literatures; (H) Comparison of capital expenditure (CAPEX), operational expenditure (OPEX), and levelized cost of lithium (LCOL) between the present PDMS‐LATP membrane and other membranes.

Although H‐cell measurements established the intrinsic lithium selectivity of the PDMS‐LATP membrane, the observed lithium flux remained limited by the weak electric‐field driving force and the high resistance of the deionized receiving phase. Electrodialysis separates ions by using an external voltage to direct their movement across selective membranes, thereby establishing ion concentration differences between adjacent compartments. Under these conditions, the measured transport behavior reflects not only the membrane itself, but also the operating configuration of the device. To evaluate membrane performance under more practical electrodialysis conditions, a customized single‐pair electrodialysis stack was therefore constructed (Figure 4A, C). The device incorporated an enlarged effective membrane area of 36 cm^2^ (Figure 4B), enabling a more representative assessment of separation performance during lithium extraction.

The ion transport behavior of the PDMS‐LATP membrane in the electrodialysis stack was first examined using single‐salt feed solutions containing 0.1 M LiCl, NaCl, or MgCl_2_, with 0.01 M KCl as the receiving electrolyte. Current‐voltage measurements revealed clear ion‐dependent transport thresholds (Figure 4D). For Li^+^, the current remained close to zero at low voltage and then increased sharply at approximately 1.5 V, indicating the onset of significant Li^+^ permeation. By contrast, Na^+^ exhibited a delayed response, with the inflection point shifting to approximately 2.5 V, consistent with a higher barrier for transport through the membrane. No measurable current response was observed for Mg^2+^ within the tested voltage range, indicating negligible permeation under these conditions. These results confirm that the membrane maintains a strong transport preference for Li^+^ under stack operation. The delayed onset of Na^+^ transport likely reflects its less favorable migration relative to Li^+^, although some Na^+^ permeation becomes possible at elevated voltage because of its relatively small ionic size and its comparable migration barrier in bulk LATP [23]. In contrast, Mg^2+^ transport remains strongly suppressed because its high dehydration energy and strongly bound hydration shell impose a substantial penalty for both interfacial entry and migration through the membrane. Based on these measurements, an operating voltage of 3 V was selected for subsequent experiments because it enabled robust Li^+^ transport while maintaining strong exclusion of competing ions. Time‐dependent concentration measurements in the electrodialysis stack further verified the selective transport behavior of the membrane (Figure 4E, F). Under identical operating conditions, Li^+^ showed the highest transport flux, whereas Na^+^ was transported much less efficiently and Mg^2+^ remained strongly rejected. We also conducted electrodialysis experiments on various other binary/mixed feed solutions, all of which demonstrated the effective selectivity of lithium ions by the PDMS‐LATP membrane (Figures S10–S13). These results are consistent with the hierarchical selectivity mechanism established above: PDMS imposes an interfacial penalty on strongly hydrated ions, while LATP provides the Li^+^‐conducting lattice pathway that governs final ion discrimination (Figure S14). The stack configuration therefore allows the intrinsic membrane selectivity to be expressed more effectively as practical separation performance.

The LATP‐PDMS membranes developed in this study demonstrate superior Li^+^/Mg^2+^ performance compared to state‐of‐the‐art membranes for Li^+^/Mg^2+^ separation (Figure 4G and Table S1). Relative to dense NASICON‐type ceramic membranes, the PDMS‐LATP membrane delivered a substantially higher lithium flux, albeit with some reduction in Li^+^/Mg^2+^ selectivity. This trade‐off arises from the composite membrane architecture. In dense ceramic membranes, tightly packed crystalline domains restrict ion transport almost exclusively to highly selective LATP lattice channels, resulting in extremely high selectivity but limited flux because of the large transport resistance associated with thick and compact structures. However, such high selectivity represents an idealized limit and may still be constrained in practice by the demanding requirements for ceramic membrane fabrication, including densification quality, microstructural defects, interfacial imperfections, and stability under aqueous operating conditions. Although the present data do not permit a rigorous partitioning of total ion flux into lattice transport and interfacial leakage components, the comparative experiments provide clear experimental evidence that non‐ideal membrane‐level transport contributes measurably to the reduced Li^+^/Mg^2+^ selectivity of the composite membrane (Figure S15). By contrast, the PDMS‐LATP membrane is much thinner and less densely packed than a sintered ceramic membrane, which lowers transport resistance and facilitates higher Li^+^ throughput. Although this less compact arrangement may permit limited transport of competing ions through interfacial regions between LATP particles and the polymer matrix, the membrane still maintains high overall selectivity because the defect‐suppressed PDMS phase effectively minimizes non‐selective leakage while preserving access to LATP‐mediated Li^+^ transport pathways.

*A techno‐economic analysis (TEA)* further highlights the practical potential of the PDMS‐LATP membrane. Under identical electrodialysis conditions using a binary feed of 0.1 M LiCl and 0.1 M MgCl_2_, the levelized cost of lithium (LCOL) for the PDMS‐50 wt.% LATP membrane was substantially lower than that of a commercial cation‐exchange membrane and a pure NASICON‐type LATP ceramic membrane (Figure 4H and Text S10). Notably, the comparatively higher LCOL observed for the NASICON membrane can be mainly attributed to two factors: (i) its lower Li^+^ flux, which necessitates a significantly larger membrane area when the analysis is normalized to a fixed lithium production target, and (ii) its higher estimated membrane cost, which is approximately 1.5 times that of the PDMS‐LATP membrane. This cost advantage of the PDMS‐LATP membrane primarily stems from its higher Li^+^ flux, which reduces the required area and overall capital expenditure, while maintaining excellent selectivity and scalability.

### 2.5 The PDMS‐LATP Membrane Sustains Selective Lithium Extraction From Authentic SWRO Brine

**FIGURE 5:**
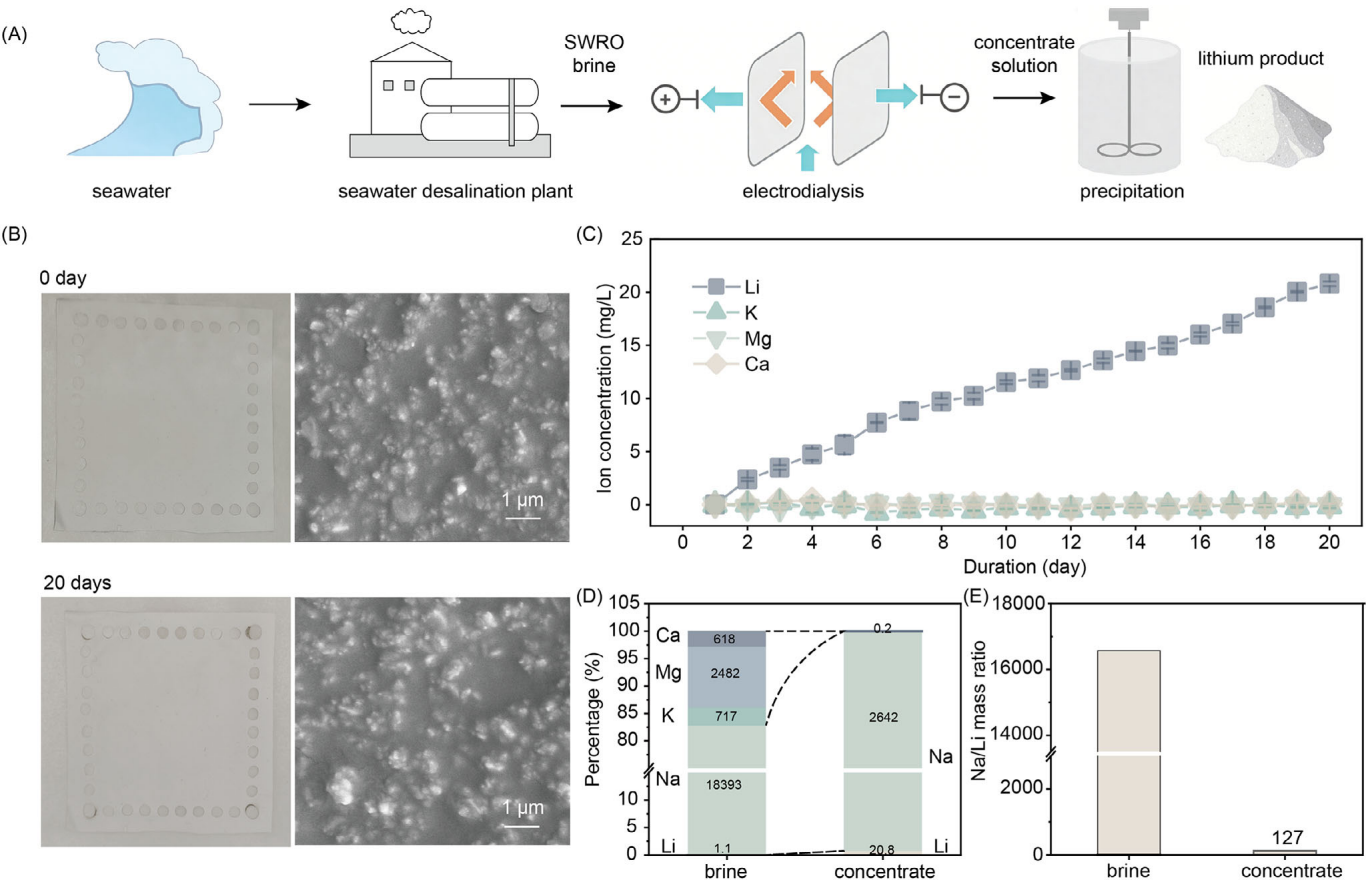
Long‐term performance and SWRO brine lithium extraction application of the PDMS‐LATP membranes. (A) Schematic illustration of the continuous lithium extraction process from SWRO brine; (B) Membrane photographs and SEM image before and after 20 days’ SWRO lithium extraction experiment; (C) Temporal variation of ion concentrations in the concentrate solution over a 20‐day extraction period; (D) Comparison of ion concentrations in seawater reverse osmosis brine before and after lithium extraction; (E) Change in the Na/Li mass ratio of the feed brine before and after the 20‐day process.

To assess practical applicability beyond model salt solutions, the PDMS‐LATP membrane was further evaluated using authentic seawater reverse osmosis brine collected from the Tseung Kwan O Desalination Plant in Hong Kong. Compared with raw seawater, SWRO brine provides a more relevant process stream for lithium recovery because it contains a concentrated inorganic ionic background with reduced organic interference, while still presenting the central challenge of extracting trace Li^+^ in the presence of overwhelming concentrations of competing cations, particularly Na^+^ and Mg^2+^ (Figure 5A) [34, 35, 36]. Electrodialysis experiments were carried out using the same stack configuration described above, with a 1 L dilute chamber containing SWRO brine and a 200 mL concentrate chamber containing 0.01 M KCl. The membrane exhibited stable and selective operation throughout the 20‐day extraction period. As shown in Figure 5C, the Li^+^ concentration in the concentrate stream increased continuously from an initially negligible value to 20.8 mg L^−1^ after 20 days. By contrast, the concentrations of K^+^, Mg^2+^, and Ca^2+^ in the concentrate showed no significant increase over the same period, indicating that the membrane retained strong rejection of these competing cations. Although Na^+^ concentration also increased and reached 2642 mg L^−1^ because of its overwhelming abundance in the feed (Figure S16), the relative enrichment of lithium remained pronounced. These results demonstrate that the PDMS‐LATP membrane can sustain selective lithium enrichment under real brine conditions. Although the present concentrate is not yet suitable for direct lithium salt production, the process successfully functions as a critical upstream enrichment step. During the 20‐day test, the 1 L feed brine was replaced daily to maintain a stable source concentration, allowing for a cumulative Li^+^ recovery of approximately 90% from the processed brine. The resulting Li‐enriched stream, with its Na/Li mass ratio dramatically reduced from 16 721 to 127 (Figure 5D, E), corresponding to more than 130‐fold enrichment of Li^+^ relative to Na^+^, would then be a much more favorable feedstock for subsequent downstream processing, such as evaporation followed by conventional carbonate precipitation. The observed Na^+^ accumulation in the concentrate likely arises from a combination of factors, including limited transport through the LATP lattice itself under the large concentration gradient and potential leakage through non‐ideal interfacial pathways or the anion exchange membrane.

The structural stability of the membrane after prolonged operation was confirmed by direct characterization. Photographs and SEM images collected before and after the 20‐day electrodialysis test showed no obvious damage, delamination, or significant change in surface morphology (Figure 5B), indicating excellent physical integrity during long‐term use. XRD analysis further showed that the crystalline structure of LATP remained unchanged after aqueous salt exposure (Figure S17), confirming the chemical stability of the ceramic phase within the composite membrane. The long‐term stability of the PDMS‐LATP membrane can be understood by comparison with pure LATP ceramic. Pure LATP membranes showed partial swelling and surface delamination after overnight immersion in salt solution (Figure S18), which is consistent with the intrinsically hydrophilic nature of phosphate‐based NASICON ceramics. The measured contact angle of pure LATP was approximately 36° (Figure S19), indicating strong affinity for water. This hydrophilicity likely facilitates water penetration into incompletely sintered regions, leading to swelling and eventual detachment. Such swelling may also impair ion transport by forcing lithium ions to undergo repeated dehydration and rehydration events within the membrane, thereby increasing transport resistance [37]. More generally, the aqueous stability of LATP ceramics should be evaluated in relation to the specific chemical and structural context, rather than treated as an unconditional material property. Previous studies suggest that prolonged exposure to water, especially under acidic or alkaline conditions [38], may alter the surface chemistry and transport behavior of LATP, whereas physical stability can depend strongly on factors such as surface condition, densification quality, and the presence of microstructural defects that promote water ingress. From this perspective, the long‐term stability demonstrated in this work should be understood as a property of the PDMS‐encapsulated composite membrane under the present operating conditions, rather than a general claim for bare LATP in all aqueous environments. In contrast, the PDMS phase forms a tight encapsulating matrix around the LATP particles, limiting water intrusion and preserving interfacial integrity under prolonged aqueous operation. This protective effect explains why the composite membrane retains both its structure and its selective transport performance under realistic high‐salinity conditions.

## 3 Discussion

In this work, we developed a flexible PDMS‐LATP membrane that translates the intrinsic lithium‐ion selectivity of a NASICON‐type solid electrolyte into a processable membrane platform for electrodialysis. By embedding LATP particles within an amorphous PDMS matrix, the membrane overcomes several key limitations of standalone LATP ceramics, including brittleness, defect sensitivity, and limited scalability, while preserving the low‐barrier Li^+^ transport characteristics of the ceramic phase. The optimized membrane achieved high Li^+^ flux together with exceptional Li^+^/Mg^2+^ selectivity under mixed‐salt conditions and maintained stable operation during prolonged extraction from real SWRO brine. More broadly, this study shows that the role of the polymer phase in lithium‐selective membranes is not merely mechanical support. Rather, the polymer governs filler dispersion, interfacial continuity, and the local solvation environment encountered by ions before entering the ceramic phase. In this sense, the PDMS‐LATP architecture represents not only a compositional optimization, but also a membrane design strategy that couples processability, interfacial control, and ion‐selective transport in a single platform.

Compared with previously reported lithium‐selective membranes and hybrid ion‐conducting membranes, the present PDMS‐LATP system offers a distinct balance between selectivity, flux, and mechanical processability. Earlier LATP‐based membranes prepared with semi‐crystalline polymer matrices often suffered from filler agglomeration, polymer‐rich bypass domains, or microvoid formation at the ceramic‐polymer interface, which limited the extent to which the intrinsic sieving function of LATP could be translated into membrane‐scale selectivity [39]. Likewise, other hybrid membranes based on lithium‐conducting ceramics combined with ion‐exchange polymers have demonstrated promising ion discrimination, but their transport behavior is frequently mediated by more complex dual‐pathway or charge‐mosaic effects, in which ion exchange domains and ceramic conduction domains coexist [40, 41]. Such architectures can enhance transport rates or reduce energy consumption, but they may also complicate the interpretation of the dominant selectivity mechanism and introduce additional trade‐offs between permselectivity, membrane swelling, and long‐term interfacial stability in high‐salinity brines. By contrast, the PDMS‐LATP membrane presented here relies on a comparatively simple and physically transparent design principle: an electrically insulating, amorphous polymer matrix suppresses nonselective aqueous ion leakage, while dispersed LATP particles provide the primary Li^+^‐conducting pathway. This design leads to a membrane that is mechanically compliant, scalable by solution casting, and highly stable in real brine environments. At the same time, our membrane does not yet reach the extreme Li^+^/Mg^2+^ selectivity sometimes reported for dense ceramic pellets [32], underscoring that the gain in manufacturability and flux is achieved with some sacrifice in the ideality of the transport pathway.

Despite these advantages, several important challenges remain before this membrane platform can be translated toward industrially relevant lithium extraction. First, the membrane still operates under a flux‐selectivity compromise inherited from its composite nature: although PDMS improves interfacial integrity and suppresses defect‐mediated leakage, the nonceramic regions and ceramic‐polymer boundaries may still permit limited transport of small competing cations, particularly Na^+^ at elevated voltage or under large concentration gradients. Additionally, because ion transport requires an applied electric field and proceeds through a discontinuous composite microstructure rather than a fully dense ceramic lattice, the energy efficiency of the overall process remains strongly dependent on stack design, current efficiency, and concentration polarization management [42, 43]. These limitations indicate that further progress will depend not only on materials optimization, but also on integrating membrane design with electrodialysis engineering.

From a theoretical perspective, the present results point to several fruitful directions for future study. Our experiments and simulations support a dual‐filter picture in which the PDMS phase modifies ion dehydration and interfacial partitioning, while LATP provides the ultimate crystallographic pathway for Li^+^ transport. However, a more predictive understanding will require multiscale modeling that bridges atomistic ion‐solvent interactions, interfacial transport barriers, and mesoscale percolation of ceramic pathways within the membrane. In particular, future work should quantify how polymer dielectric environment, interfacial free volume, and particle surface chemistry connectivity jointly determine the enthalpic and entropic contributions to ion transport. More broadly, this approach opens a path toward a new class of electrodialysis membranes that exploit solid‐electrolyte physics in soft, scalable architectures. Extending this concept to other NASICON‐type conductors [44, 45], tailored interphases, or anisotropic ceramic networks may enable membranes with higher current efficiency, lower energy consumption, and more programmable ion selectivity for lithium recovery from increasingly dilute and compositionally complex resources.

## 4 Materials and Methods

### 4.1 Materials and Chemicals

The PDMS‐LATP membrane was fabricated using Sylgard 184 (Dow Corning Corporation), consisting of a silicone elastomer base and curing agent (10:1 wt/wt ratio), with LATP powder (300 nm, Shenzhen Kejing Corporation, China) as the filler. n‐Heptane and TEOS (Macklin Reagent Corporation, China) were used as solvents and cross‐linking agents, respectively. For comparison with other polymer‐based LATP membranes, polyacrylonitrile (PAN) and poly(vinylidene fluoride‐co‐hexafluoropropylene) (PVDF‐HFP, Mw = 455 000 g mol^−1^, Sigma‐Aldrich) were dissolved in N,N‐dimethylformamide (DMF, Sigma–Aldrich). The separation performance was evaluated using inorganic salts, including lithium chloride (LiCl), sodium chloride (NaCl), potassium chloride (KCl), magnesium chloride hexahydrate (MgCl_2_·6H_2_O), and calcium chloride (CaCl_2_), all purchased from Aladdin Reagent Corporation (China).

### 4.2 Membrane Fabrication

A well‐dispersed LATP‐PDMS membrane was prepared via a solution‐based synthesis approach. First, a predetermined amount of LATP powder was dispersed in 4 mL of heptane under continuous stirring. To improve interfacial compatibility between the ceramic filler and the polymer matrix, TEOS was added as a coupling agent at a fixed weight ratio (LATP:TEOS = 10:1), and the mixture was stirred for 12 h to ensure complete surface modification of LATP particles. The modified LATP suspension was then mixed with 2 g of PDMS prepolymer and 0.2 g of curing agent, followed by 2 h of vigorous stirring at room temperature. By varying the LATP/PDMS weight ratio (10–70 wt.%), a series of composite membranes with different filler loadings was obtained. The homogeneous slurry was cast onto glass substrates and thermally cured at 90°C for 3 h. The fabricated membrane can easily peel from the glass plate.

### 4.3 Electrodialysis Separation Performance Test

A custom three‐membrane/four‐compartment electrodialysis cell was constructed using customized components, with a membrane stack comprising ASTOM (Japan) AEMs (ASE) and CEM (CSE) (6 × 6 cm effective area), where the commercial CEM was alternatively replaced with a self‐made PDMS‐LATP membrane for comparative testing. Each membrane was sealed with rubber gaskets to prevent leakage, while stainless steel (anode) and graphite (cathode) electrodes were positioned at the outer ends, flanking the electrode rinse compartments separated by AEMs. The inner feed and receiving channels, interfacing with the central CEM (or PDMS‐LATP alternative), facilitated solution flow, with the entire assembly secured under compression via bolting.

In the electrodialysis experiment, 500 mL of 0.1 M salt solution and 0.01 M KCl were pumped into the dilute chamber and concentrate chamber, respectively, at a volumetric flow rate of 90 mL/min. A 0.3 M Na_2_SO_4_ electrode rinse solution was continuously recirculated from the same reservoir at a flow rate of 100 mL/min. A constant potential of 3 V was applied for 1 h. The concentrate solution was sampled every 10 min, and the cation concentration was analyzed using ion chromatography (Thermo Scientific Dionex ICS‐5000+). The flux of ions (*J_i_*) was calculated according to

(1) $$J_{i}=\frac{\Delta C_{i}V}{A\Delta t}$$

where Δ*C_i_* represents the change in ion concentration in concentrate solution, V is the volume of concentrate solution, A is the effective membrane surface (36 cm^2^), and Δ*t* is the time duration. The selectivity of the membrane to lithium over other cations (*S*_*Li*/*i*_) is defined as the ratio of ion flux normalized by their initial concentrations.

(2) $$S_{Li/i}=\frac{P_{Li}}{P_{i}}\;\frac{C_{i}}{C_{Li}}$$

where *P_Li_* and *P_i_* are the permeation rates of lithium and other cations, *C_Li_* and *C_i_* are the feed concentrations of Lithium and other cations, respectively.

### 4.4 Calculations of Energy Barriers

The energy barrier for lithium transport across the membrane was determined by evaluating the temperature dependence of membrane conductivity. A temperature gradient was established at 25, 30, 35, 40, and 45°C. The solution temperature was monitored and controlled using a hotplate probe. Transition state theory was employed to calculate the energy barriers for lithium ion transport in the membrane. The relationship between ionic conductivity and the entropy barrier (Δ*S*) and enthalpy barrier (Δ*H*) was derived using Equation (3).

(3) $$ln\sigma =\ln\left(\frac{\lambda^{2}k_{B}F^{2}C}{Rh}\right)\;-\frac{\Delta H}{RT}+\frac{\Delta S}{R}$$

where σ (S·m^−1^) refers to the ionic conductivity, ℎ is Planck's constant (6.626 × 10^−34^ J·s), *k_B_* is the Boltzmann constant (1.381 × 10^−^^2^ ^3^ J·K^−^^1^), 𝑅 is the ideal gas constant (8.314 J·mol^−1^·K^−1^), *F* refers to the Faraday constant (96485.333 C·mol^−1^), *N_A_* refers to the Avogadro constant (6.022 × 10^23^ mol^−1^), λ refers to the distance between equilibrium positions (The average Li^+^ jump length in the LATP was determined to be 4.0 Å based on crystal structure analysis, reflecting migration between lattice and interstitial sites [32]), and T (K) refers to the temperature.

The concentration of lithium inside the SSE was approximated according to the stoichiometry Li_1.3_Al_0.3_Ti_1.7_(PO_4_)_3_. Specifically, the lithium ion concentration is determined according to the molecular weight of the LATP structure (*MW*), and the mass (*m*), area (*A_m_*), and thickness (δ_*m*_) of a fresh SSE membrane coupon.

(4) $$C=\frac{m}{MW}\;\times \frac{1.3\;mol\;Li}{1\;mol\;SEE}\times \frac{1}{A_{m}\delta_{m}}$$

Detailed methods of experiment and simulation data are provided in Supplementary Information.

## Author Contributions

Conceptualization: AKA, XW, Methodology: XW, JS, Investigation: MWB, XL, MK, JK, HC, PHLS, YW, ZL, Supervision: AKA, JCHL, ZL, Writing – original draft: XW, Writing – review & editing: AKA, JCHL, ZL

## Funding

This work was supported by the Research Grants Council of Hong Kong through Research Fellow Scheme (Project No. RFS2223‐1S04) and General Research Fund (Project No. 11218122) Additional support was provided by the Hong Kong Innovation and Technology Commission (ITC‐CNERC14EG03).

## Conflicts of Interest

The authors declare no conflict of interest.

## Supporting information




**Supporting File**: advs76649‐sup‐0001‐SuppMat.docx.

## Data Availability

The data that support the findings of this study are available from the corresponding author upon reasonable request.
