# Supplementary material for: Ion Superhighways in a Hierarchical Polymer‐Ceramic Membrane Enable Rapid and Selective Lithium Extraction
Source: Adv Sci (Weinh). 2026 Jul 20;13(56):e76649. doi: 10.1002/advs.76649 (PMC13383148; doi:10.1002/advs.76649)
Supplement: Supplementary file 1 [file ADVS-13-e76649-s001.docx]

**Supplementary information**

**Ion superhighways in a hierarchical polymer–ceramic membrane enable rapid and selective lithium extraction**

Xinxin Wei^a^, Jiawei Sun^b^, Min Wei Boey^a^, Xiaolu Li^a^, Minji Kim^c^, Junghwan Kim^c^, Hongjiang Chen^a^, Patrick H.-L. Sit^a^, Yixiang Wang^d^, Zhigang Li^d^ , Ze-Xian Low^e*^, Jason Chun-Ho Lam^a*^, and Alicia Kyoungjin An^b*^

^a^ School of Energy and Environment, City University of Hong Kong, 83 Tat Chee Avenue, Kowloon, Hong Kong, 999077, China

^b^ Department of Chemical and Biological Engineering, The Hong Kong University of Science and Technology, Hong Kong, 999077, China

^c^ Department of Chemical and Biomolecular Engineering, Yonsei University, Seoul, 03722, Republic of Korea

^d^ Department of Mechanical and Aerospace Engineering, The Hong Kong University of Science and Technology, Hong Kong, 999077, China

^e^ State Key Laboratory of Materials-Oriented Chemical Engineering, National Engineering Research Center for Special Separation Membrane, Nanjing Tech University, Nanjing, 211816, China

*Corresponding author. Email: nicholas.low@njtech.edu.cn, jason.lam@cityu.edu.hk, alicia.kjan@ust.hk

**Text S1. Membrane fabrication**

**1.1 PMDS-LATP membrane**

A well-dispersed LATP-PDMS membrane was prepared via a solution-based synthesis approach. First, a predetermined amount of LATP powder was dispersed in 4 mL of n-heptane under continuous stirring. To improve interfacial compatibility between the ceramic filler and the polymer matrix, TEOS was added as a coupling agent at a fixed weight ratio (LATP:TEOS = 10:1), and the mixture was stirred for 12 h to ensure complete surface modification of LATP particles. The modified LATP suspension was then mixed with 2 g of PDMS prepolymer and 0.2 g of curing agent, followed by 2 h of vigorous stirring at room temperature. By varying the LATP/PDMS weight ratio (10–70 wt%), a series of composite membranes with different filler loadings were obtained. The homogeneous slurry was cast onto glass substrates and thermally cured at 80°C for 10 h, yielding freestanding membranes with a uniform thickness.

**1.2 PAN-LATP and PVDF-LATP membrane**

The PAN/PVDF-LATP membranes were fabricated using a solution casting technique. Polymer solutions were prepared by dissolving PAN or PVDF (10 wt% each relative to solvent) in N,N-dimethylformamide (DMF) at 60°C with constant mechanical stirring until complete dissolution was achieved. LATP ceramic filler (10 wt% relative to solvent) was subsequently incorporated into the polymer solution and dispersed uniformly through probe ultrasonication. The excellent compatibility between DMF and LATP particles facilitated homogeneous filler distribution without significant agglomeration. The resulting suspension was cast onto clean glass substrates using a doctor blade, followed by 48 hours of drying at room temperature in a fume hood.

**1.3 LATP ceramic membrane**

LATP particles were placed in a 2 cm diameter steel mold and compressed using a tablet press at 30 MPa. The thickness of the resulting LATP ceramic membrane was controlled at approximately 1 mm by adjusting the mass of LATP particles. The pellet was then annealed in a muffle furnace at 870°C with a heating rate of 5°C/min and cooled to room temperature before taking out.

**Text S2. Membrane characterization**

The surface morphologies of the membranes were characterized using a Scanning Electron Microscope (SEM, Carl-Zeiss) and a Field Emission Scanning Electron Microscope (FESEM, FEI JOEL-7800F). Elemental mapping was also conducted on the FE-SEM. The mechanical strength of the membrane was evaluated by tensile testing using a material testing instrument (Lloyd LS1, AMETEK, U.S.). The water contact angle of the membrane was measured with a Krüss EASYDROP contact angle system (Germany) by depositing a deionized water droplet onto the membrane surface via a syringe with a thin needle. Images of the water droplets were captured using a video-digitizer board, and the contact angle was determined by the sessile drop method with DSA1 software. All reported values represent the average of three independent measurements.

**Text S3. Electrochemical testing and sample analysis**
Electrolysis was performed in a custom H-cell separated by a membrane. The cathode and anode compartments each contained a platinum wire electrode (effective area 2 cm^2^ per electrode), sealed using silicone rubber O-rings to ensure airtight conditions. A constant voltage of 2.0 V was applied between the electrodes using an Metrohm Autolab potentiostat. Electrolyte samples (~1 mL) were extracted hourly from the anode compartment using a gas-tight syringe and immediately.

**Text S4. Ion concentration analysis**
Collected samples were filtered (0.22 µm nylon membrane) and analyzed for cation concentration using Ion Chromatography (Thermo Scientific Dionex ICS-5000+). Separation was achieved on a cation-exchange column (4 mm diameter, Dionex IonPac CS12A) using an isocratic 20 mM methanesulfonic acid eluent at 1.0 mL/min, complemented by a CERS 500 (4 mm) suppressor. Detection was via conductivity, and quantification was performed using external calibration standards.

**Text S5. Ion conductivity**

The through-plane membrane resistance was measured via a two-electrode electrochemical impedance spectroscopy (EIS) technique using a Metrohm AUTOLAB potentiostat, with an AC bias of 10 mV and a frequency range of 1 MHz to 100 Hz. Membranes were saturated with a 0.1 M aqueous electrolyte (ionic strength: *I* = 0.1 M) and sandwiched between two Pt electrodes for testing under elevated temperatures. The ionic conductivity of the membranes was calculated using the following equation:

$$\begin{aligned} \sigma=\frac{L}{RA}\#\left( 1 \right) \end{aligned}$$

where 𝜎 is the ion conductivity, L is the distance between a pair of electrodes, A is the effective area of membrane, and R is membrane resistance (Ω), obtained from Nyquist plot fitting using the equivalent circuit model.

**Text S6. Determination of energy barriers**

Transition state theory was applied to determine the energy barriers for lithium-ion transport in the membrane. The relationships between the ionic conductivity and the entropy barrier (△S) and enthalpy barrier (△H) can be calculated via Eq. (2).

$$\begin{aligned} ln\sigma=\ln\left( \frac{{\lambda^{2}k_{B}F}^{2}C}{Rh} \right)-\frac{\Delta H}{RT}+\frac{\Delta S}{R}\#\left( 2 \right) \end{aligned}$$

where $\sigma$ (s·m^-1^) refers to the ionic conductivity, ℎ is Planck’s constant (6.626×10^-34^ J·s), $k_{B}$ is Boltzmann constant (1.381×10^-23^ J·K^-1^), 𝑅 is the ideal gas constant (8.314 J·mol^-1^·K^-1^), $F$ refers to the Faraday constant (96485.333 C·mol^-1^), $N_{a}$ refers to the Avogadro constant (6.022 × 10^23^ mol^-1^), $\lambda$ refers to the distance between equilibrium positions (The average Li^+^ jump length in the LATP was determined to be 4.0 Å based on crystal structure analysis, reflecting migration between lattice and interstitial sites(*1*)), and T (K) refers to the temperature.

The concentration of lithium inside the SSE was approximated according to the stoichiometry Li_1.3_Al_0.3_Ti_1.7_(PO_4_)_3_. Specifically, the lithium ion concentration is determined according to the molecular weight of the LATP structure ($MW$), and the mass ($m$), area ($A_{m}$), and thickness ($\delta_{m}$) of a fresh SSE membrane coupon.

$$\begin{aligned} C=\frac{m}{MW}\times\frac{1.3 mol Li}{1 mol SEE}\times\frac{1}{A_{m}\delta_{m}}\#\left( 3 \right) \end{aligned}$$

**Text S7. Electrodialysis cell design**

A custom three-membrane/four-compartment electrodialysis cell was constructed using DESALT-customized components. The membrane stack comprised ASTOM JAPAN anion-exchange membranes (AEMs) and a cation-exchange membrane (CEM) with an effective area of $6\times6 cm^{2}$, where the commercial CEM was alternatively replaced with the self-fabricated PDMS-LATP membrane for comparative testing. Each membrane was sealed with rubber gaskets to prevent leakage. Platinum and graphite electrodes were positioned at the outer ends of the cell as the anode and cathode, respectively, flanking the electrode-rinse compartments separated by AEMs. The inner dilute and concentrate channels were adjacent to the central CEM (or PDMS-LATP membrane), and the entire assembly was secured under compression by bolting.

During electrodialysis operation, a $0.3 M Na_{2}SO_{4}$solution was used as the electrode-rinse electrolyte. At the anode and cathode, water electrolysis may occur according to:

$$\text{Anode:}\text{ }2H_{2}O\to O_{2}+4H^{+}+4e^{-}$$

$$\text{Cathode:}\text{ }2H_{2}O+2e^{-}\to H_{2}+2OH^{-}$$

These reactions can locally generate acidic and alkaline species in the respective electrode compartments. However, in the present setup, the effluents from both electrode compartments were returned to the same external reservoir, which promotes rapid neutralization within the electrode loop and minimizes net pH disturbance to the process streams.

Possible effects associated with electrodialysis operation, including electrode reactions, pH variation, and concentration polarization, were assessed by monitoring the current response and the pH of the dilute and concentrate streams during operation. Under the present conditions, no significant pH drift was observed in the process streams, indicating that electrode-side acid/base generation did not measurably perturb the bulk feed and receiving solutions. In addition, continuous recirculation of both the dilute and concentrate streams was applied to mitigate concentration polarization.

**Text S8. Energy consumption**

**8.1** **Calculation of Li^+^ current efficiency**

The Li^+^ current efficiency was calculated according to:

$$\eta_{\mathrm{Li}}=\frac{zFJ_{\mathrm{Li}}}{j_{\mathrm{avg}}}$$

where $\eta_{\mathrm{Li}}$is the current efficiency for Li^+^ transport, $z$is the charge number of Li^+^, $F$ is the Faraday constant $\left( 96485 C mol^{-1} \right)$, $J_{\mathrm{Li}}$is the measured Li^+^ flux, and $j_{\mathrm{avg}}$ is the average current density during electrodialysis.

The measured Li^+^ flux was:

$$J_{\mathrm{Li}}=2.689\times10^{-4}\mathrm{mol}m^{-2} s^{-1}$$

The average current density obtained from the current density-time profile was:

$$j_{\mathrm{avg}}=2.90 mA cm^{-2}=29.01 A m^{-2}$$

Thus, the Li^+^ current efficiency was calculated as:

$$\eta_{\mathrm{Li}}=\frac{1\times96485\times2.689\times10^{-4}}{29.01}=89.4\%$$

**8.2 Calculation of energy consumption normalized to recovered lithium**

The energy consumption normalized to recovered lithium was calculated according to:

$$E_{\mathrm{Li}}=\frac{Vj_{\mathrm{avg}}}{J_{\mathrm{Li}}}$$

where $E_{\mathrm{Li}}$is the electrical energy consumption per mole of recovered Li, $V$is the applied voltage, $j_{\mathrm{avg}}$is the average current density, and $J_{\mathrm{Li}}$is the measured Li^+^ flux.

Using an applied voltage of:

$$V=3 V$$

the normalized energy consumption was obtained as:

$$E_{\mathrm{Li}}=323.7 kJ mol^{-1}=89.9 Wh mol^{-1}\mathrm{Li}$$

**Text S9.** **Density functional theory Computational details**

Density functional theory (DFT) was performed using Vienna ab initio simulation software package (VASP)(*2*–*4*). The projection enhanced wave method was employed(*5*, *6*). The generalized gradient approximation (GGA) and Perdew-Burke-Ernzerhof (PBE) exchange function are used throughout the calculation(*7*) . The CI-NEB method is employed to calculate the transition state and ion migration barrier(*8*). The cutoff energy of the plane wave was set at 600 eV. K-point of Brillouin region integration is 3 × 3 × 1 grid centered at point Gamma. The convergence criteria for energy and force calculation are set as 10^−5^ eV/atom and 0.01 eV/ Å during relaxation. All frequency calculations set convergence criteria for energy and force calculations as 10^−7^ eV/atom and 0.01 eV/ Å.

Structure of LiTi_2_ (PO_4_)_3_ is taken from experiment data (*9*). The original structure contains 108 atoms with cell parameters as a=b=8.511Å, c= 20.84300Å α=β=90, γ=120. We build the LATP structure, starting and final position of the migration ion the same as previous studies, in which two Ti atoms in the unit cell are replaced by Al (*10*). Structures are then relaxed with all atoms free to move while cell parameters fixed. All three types of ions are considered to take the same migration pathway in our study.

**Text S10. Techno-Economic Analysis**

A techno-economic analysis (TEA) was conducted as a case-study–level evaluation to assess the relative economic performance of the PDMS-LATP membrane in comparison with a commercial cation-exchange membrane and a NASICON-type LATP ceramic membrane under identical electrodialysis conditions. A conventional ED stack configuration consisting of AEM–Li-selective membrane–AEM was assumed, and all non-membrane components (electrodes, spacers, frames, and peripheral equipment) were treated identically across all cases.

The operating conditions were fixed for all membranes, including a binary feed solution of 0.1 M LiCl and 0.1 M MgCl_2_, a constant cell voltage of 3 V, 8,500 operating hours per year, and a membrane lifetime of 3 years. The annual lithium production target was set to 1,000 kg Li per year, and the analysis was intended to capture relative cost trends driven by membrane performance, rather than to represent a fully optimized industrial plant design.

**10.1 Flux-based membrane area determination**

The required membrane area was determined directly from experimentally measured Li⁺ flux values. The annual lithium production per unit membrane area was calculated as

$$\begin{aligned} P_{\mathrm{Li}}=J_{\mathrm{Li}}\times t_{\mathrm{op}}\times\frac{M_{\mathrm{Li}}}{1000}\#\left( 5 \right) \end{aligned}$$

where $J_{\mathrm{Li}}$is the Li⁺ flux (mmol m^-2^ h^-1^), $t_{\mathrm{op}}$is the annual operating time (h yr^-1^), and $M_{\mathrm{Li}}$is the molar mass of lithium.

The total membrane area required to meet the fixed lithium production target was then calculated as

$$\begin{aligned} A=\frac{P_{\mathrm{Li},\mathrm{target}}}{P_{\mathrm{Li}}}\#\left( 6 \right) \end{aligned}$$

This flux-based sizing approach directly links intrinsic membrane performance to system-level economics. Membranes exhibiting higher Li^+^ flux require a smaller membrane area, resulting in lower capital expenditure and reduced area-dependent operating costs.

**10.2 Electrical current and energy consumption**

The total electrical current was estimated from the molar fluxes of Li^+^ and Mg^2+^ ions using Faraday’s law, accounting for their respective charge numbers:

$$\begin{aligned} I=F\left( J_{\mathrm{Li}}+2J_{\mathrm{Mg}} \right)A\#\left( 7 \right) \end{aligned}$$

where $F$is Faraday’s constant, $J_{\mathrm{Mg}}$is the Mg^2+^ flux, and $A$is the total membrane area.

Annual electricity consumption was calculated as

$$\begin{aligned} E=V\times I\times t_{\mathrm{op}}\#\left( 8 \right) \end{aligned}$$

with a fixed cell voltage $V=3$V. The electricity cost was obtained by multiplying the annual energy consumption by a unit electricity price of 0.13 USD kWh^-1^(*11*).

**10.3 Capital expenditure and annualization**

The capital expenditure (CAPEX) was composed of the membrane module cost, stack assembly cost, and peripheral equipment cost. The membrane module cost included the cost of two AEMs and one Li-selective membrane per unit area, while stack and peripheral costs were estimated as fixed multiples of the membrane module cost (1.5× and 0.5×, respectively), following conventional ED economic assumptions(*12*).

The total CAPEX was annualized using a capital recovery factor (CRF):

$$\begin{aligned} \mathrm{CRF}=\frac{r(1+r)^{n}}{(1+r)^{n}-1}\#\left( 9 \right) \end{aligned}$$

where $r=7\%$ is the discount rate and $n=20$years is the assumed system lifetime.

**10.4 Operating expenditure**

Operating expenditure (OPEX) included electricity consumption, membrane replacement costs (based on a membrane lifetime of 3 years), and maintenance costs, which were assumed to be 10% of the annualized CAPEX(*12*). In addition, a minor Mg-related downstream handling cost was included to reflect the impact of Mg^2+^ co-transport on product purity requirements.

**10.5 Estimation of Mg-related downstream cost**

To estimate the capital expenditure (CAPEX) for the auxiliary unit operations used to quantify the Mg-related downstream handling cost, we adopted a bare module costing approach(*11*). For a jacketed, agitated CSTR, the reference purchase cost $C_{p}^{o}$ was calculated as a function of the reactor volume $V$(m^3^) using:

$$\begin{aligned} {log}_{10}\left( C_{p}^{o} \right)=K_{1}+K_{2}{log}_{10}\left( V \right)+K_{3}\left[ {log}_{10}\left( V \right) \right]^{2}\#\left( 10 \right) \end{aligned}$$

where the cost coefficients for a jacketed agitated reactor were $K_{1}=4.1052$, $K_{2}=0.5320$, and $K_{3}=-0.0005$. This correlation is applicable over $0.1\leq V\leq35$m^3^. For capacities outside the valid range, the purchase cost was extrapolated using the six-tenths rule:

$$\begin{aligned} C_{p}^{o}\left( V \right)=C_{p}^{o}\left( V_{\mathrm{ref}} \right)\left( \frac{V}{V_{\mathrm{ref}}} \right)^{0.6}\#\left( 11 \right) \end{aligned}$$

where $V_{\mathrm{ref}}=35$m^3^ was used when $V>35$m^3^, and $V_{\mathrm{ref}}=0.1$m^3^ was used when $V<0.1$m^3^.

The bare module cost for the CSTR was then obtained using a bare module factor $F_{BM}$:

$$\begin{aligned} C_{BM}=F_{BM}\text{ }C_{p}^{o}\#\left( 12 \right) \end{aligned}$$

where $F_{BM}=4.0$ for the CSTR in this study. The same approach was applied to other equipment where relevant (e.g., separation/filtration units) using their corresponding capacity measures and cost coefficients.

Finally, the total installed CAPEX for the Mg-related module was calculated by aggregating the bare module costs across all included equipment as:

$$\begin{aligned} \mathrm{CAPEX}=1.18C_{BM}+0.50C_{BM}^{o}\#\left( 13 \right) \end{aligned}$$

where $C_{BM}$is the bare module cost of unit, and $C_{BM}^{o}$denotes the auxiliary cost term returned by the same equipment cost function in the economic module. In the present implementation, the equipment cost function returns identical values for $\left( C_{BM},C_{BM}^{o} \right)$for CSTR, consistent with the adopted cost structure.

Operating costs included electricity consumption, NaOH reagent usage, maintenance, and labor. A by-product credit was applied assuming the sale of Mg(OH)_2_ at a unit price of 0.46 USD kg^-1^(*11*).

The resulting net annual cost was normalized by the annual Mg removal rate to obtain a unit Mg removal cost, which was then incorporated into the TEA as a secondary correction term.

**10.6 Definition of levelized cost of lithium**

The levelized cost of lithium (LCOL) was defined as the ratio of the total annualized cost to the target annual lithium production:

$$\begin{aligned} \mathrm{LCOL}=\frac{\mathrm{CAPEX}\times\mathrm{CRF}+\mathrm{OPEX}}{P_{\mathrm{Li},\mathrm{target}}}\#\left( 14 \right) \end{aligned}$$

where CAPEX is the total capital expenditure, OPEX is the total annual operating expenditure, and $P_{\mathrm{Li},\mathrm{target}}$is the annual lithium production target (1,000 kg Li yr^-1^).

The capital recovery factor (CRF) was used to annualize the capital cost and is given by:

$$\begin{aligned} \mathrm{CRF}=\frac{r(1+r)^{n}}{(1+r)^{n}-1}\#\left( 15 \right) \end{aligned}$$

where $r$is the discount rate (7%) and $n$is the system lifetime (20 years).

**10.10 Summary of techno-economic results and comparison metrics**

To facilitate direct comparison among different membrane types, the key membrane performance parameters and techno-economic indicators were summarized in a single table, which includes experimentally measured membrane properties as well as derived economic metrics under identical electrodialysis operating conditions.

| Item | ASTOM | NASICON | This work |
| --- | --- | --- | --- |
| **Material Performance & Flux** |  |  |  |
| Li Flux (mmol/m^2^/h) | 700 | 360.18 | 914.90 |
| Mg Flux (mg/m^2^/h) | 70 | 0.018 | 0.629 |
| Li^+^/Mg^2+^ Selectivity | 10 | 20000 | 1454.6 |
| Membrane Cost Est. ($/m^2^) | 300 | 150 | 100 |
| Required Area (m^2^) | 24.22 | 47.07 | 18.53 |
| **Annual Output & CAPEX** |  |  |  |
| Li Production (kg/m^2^/year) | 41.293 | 21.247 | 53.970 |
| Membrane CAPEX ($) | 14,288.14 | 20,708.87 | 7,226.26 |
| Stack CAPEX ($) | 21,432.20 | 31,063.30 | 10,839.39 |
| Peripheral CAPEX ($) | 10,716.10 | 15,531.65 | 5,419.70 |
| Total CAPEX ($) | 46,436.44 | 67,303.81 | 23,485.35 |
| Annualized CAPEX ($/year) | 4,383.27 | 6,353.00 | 2,216.85 |
| **Operating Costs (OPEX)** |  |  |  |
| Electricity Cost ($/year) | 1,807.36 | 1,506.28 | 1,508.20 |
| Maintenance ($/year) | 438.33 | 635.30 | 221.69 |
| Membrane Replacement ($/year) | 4,762.71 | 6,902.96 | 2,408.75 |
| Mg Treatment Cost ($/year) | 1,913.58 | 0.96 | 13.16 |
| Total OPEX ($/year) | 8,921.98 | 9,045.49 | 4,151.79 |
| **Total Cost & Lithium Cost** |  |  |  |
| Total Annual Cost ($/year) | 13,305.25 | 15,398.50 | 6,368.65 |
| Lithium Cost of Production ($/kg Li) | 13.31 | 15.40 | 6.37 |

**Fig. S1.** FTIR characterization, wettability, and surface morphology of unmodified LATP-PDMS composite membranes. (A) FTIR spectra. (B) Water contact angles measured on the membrane surfaces. (C) Surface SEM images.

Compared with the membrane containing TEOS-modified LATP, the membrane prepared with unmodified LATP exhibits generally stronger PDMS characteristic bands, including those near 1416, 1258, and 1090-1020 cm^-1^. This suggests a higher relative contribution of the PDMS phase in the probed region, which may be associated with poorer LATP/PDMS interfacial compatibility, less uniform filler dispersion, and local PDMS enrichment in the unmodified membrane. After TEOS modification, the formation of a siloxane-rich interfacial layer likely improves polymer–ceramic interactions and reduces the relative intensity of PDMS-related absorption bands.

The unmodified LATP-PDMS membrane exhibits a clear difference in water contact angle between its two surfaces, indicating asymmetric surface composition and morphology. The LATP-rich and rougher side shows a lower contact angle of 91°, suggesting reduced hydrophobicity due to greater inorganic particle exposure, whereas the opposite side shows a higher contact angle of 108°, consistent with a more PDMS-dominated surface. This asymmetry reflects the limited compatibility between unmodified LATP and PDMS.

**Fig. S2.** SEM characterization of PDMS-LATP membranes with varying filler loadings: (A) PDMS-10%LATP, (B) PDMS-30%LATP, and (C, D) PDMS-70%LATP at different magnifications

**Fig. S3.** (A) Cross-sectional EDS mapping over the full membrane thickness; (B) local part of membrane cross-section.

**Fig. S4.** SEM characterization of composite membranes: Surface morphology of (A) PAN-50%LATP and (B) PVDF-50%LATP; Cross-sectional views of (C) PAN-50%LATP and (D) higher magnification image showing microstructural details

**Fig. S5.** Mechanical strength of various polymer-based membranes

**Fig. S6.** SEM photograph of LATP ceramic membrane (A) front and (B) cross-section

**Fig. S7.** Comparison of dense LATP and PDMS-LATP membranes in H-cell tests using a mixed-salt solution (0.5 M).

**Fig. S8.** Temperature-dependent ion transport through the PDMS-LATP membrane and the corresponding apparent activation energies.

**Fig. S9.** Nyquist plot of (A) LATP membrane and (B) PDMS-LATP membrane in 0.1 M solutions of LiCl; (C) Arrhenius plots of lithium conductivity

**Fig. S10.** Ion fluxes and Li⁺/Mⁿ⁺ selectivity of a commercial cation exchange membrane in single-salt solutions (0.1 M LiCl, NaCl, and MgCl_2_) under electrodialysis conditions

**Fig. S11.** Ion flux and selectivity coefficients of Li^+^ over Na^+^ and Mg^2+^ in binary equimolar (0.1 M:0.1 M) mixtures of LiCl/NaCl and LiCl/MgCl_2_ using the PDMS-LATP membrane in electrodialysis

**Fig. S12.** Effect of feed Mg^2+^/Li^+^ molar ratio (1:1 to 10:1, fixed Li^+^ concentration of 0.1 M) on Li^+^ and Mg^2+^ fluxes and Li^+^/Mg^2+^ selectivity in binary LiCl-MgCl_2_ mixtures

**Fig. S13.** Competitive electrodialysis performance of the PDMS-LATP membrane in a 0.5 M mixed-salt feed.

**Fig. S14.** SEM photographs and EDX mapping of membrane surfaces after exposure to MgCl_2_

Energy-dispersive X-ray spectroscopy (EDX) mapping of magnesium (Mg) distribution on the surfaces of (a) LATP ceramic membrane, (b) commercial cation exchange membrane (CEM, ASTOM, Japan), and (c) PDMS-LATP membrane (50 wt% LATP loading) after immersion in 0.1 M MgCl₂ solution for 24 hours. The LATP ceramic membrane exhibits minimal Mg signal, attributed to the adsorption of Mg²⁺ ions by negatively charged phosphate groups within its rhombohedral structure. In contrast, the commercial CEM displays significant Mg accumulation, reflecting the high affinity of its charged functional groups for Mg^2+^ ions. The PDMS-LATP membrane shows negligible Mg presence, owing to the low dielectric constant and hydrophobic nature of the PDMS matrix, which limits Mg^2+^ adsorption while maintaining selective Li⁺ conduction pathways enabled by uniformly dispersed LATP particles. These results highlight the superior ion-sieving performance of PDMS-LATP membranes for selective lithium extraction.

**Fig. S15.** Effect of LATP surface modification on lithium-selective transport in PDMS-LATP membranes under electrodialysis conditions.

**Fig. S16.** Increase in ion concentrations (including sodium) over a 20-day period

**Fig. S17.** XRD patterns of PDMS-LATP composite membrane before and after soaking in 0.1M LiCl aqueous solution for around 24 hours

The XRD patterns of the PDMS-LATP composite membrane before and after soaking in 0.1M LiCl aqueous solution for around 24 hours exhibit nearly identical diffraction peaks, confirming the structural stability of LATP within the PDMS matrix. The absence of new phases, peak shifts, or intensity variations suggests that LATP maintains its crystalline integrity without chemical degradation or reaction with PDMS under electrodialysis conditions. This stability is critical for the long-term performance of the composite membrane in electrochemical applications, ensuring consistent ion transport and mechanical durability

**Fig. S18.** Visible swelling and delamination of LATP ceramic membrane after soaking in 0.1M LiCl aqueous solution for around 24 hours

**Fig. S19.** Contact angle of LATP ceramic membrane

**Table S1.** Comparison of Lithium flux and selectivity

| Lithium flux | Li^+^/Mg^2+^  selectivity | Solution | Material | Reference |
| --- | --- | --- | --- | --- |
| 123 | 36 | 0.1M mixed solution | COF | Wu et al., Adv. Mater., 2025 |
| 210 | 1352 | 0.1M mixed solution | COF | Meng et al., Proc. Natl. Acad. Sci. U.S.A., 2024 |
| 40 | 35.8 | 0.1M mixed solution | COF | Sheng et al., Adv. Mater., 2021 |
| 200 | 81 | 0.1M mixed solution | COF | Meng et al., Nat. Water, 2025 |
| 20 | 12 | 0.1M mixed solution | GO | Xi et al., J. Memb. Sci., 2018 |
| 150 | 5.4 | 0.1M mixed solution | MOF | Xu et al., Sci. Adv., 2024 |
| 270 | 9.8 | 0.1M mixed solution | MOF | Xu et al., Sci. Adv., 2024 |
| 320 | 13 | 0.1M mixed solution | MOF | Xu et al., Sci. Adv., 2024 |
| 66 | 26.7 | 0.2M mixed solution | Mxene | Lu et al., Angew. Chem., 2021 |
| 1400 | 8.75 | 0.2M mixed solution | Mxene | Ren et al., J. Phys. Chem. Lett., 2015 |
| 360.1786486 | 20000 | 0.1M single salt solution | NASICON | Li et al., Adv. Funct. Mater., 2025 |
| 200 | 25283.7 | 0.1M mixed solution | NASICON | Fan et al., Adv. Sci., 2024 |
| 150 | 150 | 0.1M mixed solution | PIM | Yang et al., Nat. Water, 2025 |
| 330 | 8 | 0.1M mixed solution | polymer | Ul Afsar et al., Sep. Purif. Technol., 2021 |
| 1200 | 4.61 | 0.1M mixed solution | polymer | Zhang et al., AIChE J., 2022 |
| 36 | 634 | 1M mixed solution | polymer | Wen et al., Adv. Funct. Mater., 2016 |
| 14 | 21.2 | 1M mixed solution | polymer | Wang et al., Nat. Commun., 2018 |
| 80 | 100 | 0.1M mixed solution | polymer | Xu et al., J. Am. Chem. Soc., 2022 |
| 1870 | 31.83 | 0.1M mixed solution | polymer | Wang et al., Water Res., 2025 |
| 600 | 30000 | 0.1M mixed solution | NASICON | Patel et al., Sci. Adv., 2025 |
| 50 | 40 | 0.05M mixed solution | LATP-polymer | Seo et al., J. Memb. Sci., 2025 |
| 914.8978 | 1454.6 | 0.1M mixed solution | LATP-polymer | This work |

References:

1.	S. K. Patel, A. Iddya, W. Pan, J. Qian, M. Elimelech, Approaching infinite selectivity in membrane-based aqueous lithium extraction via solid-state ion transport. *Science Advances* **11**, 9823 (2025).

2.	G. Kresse, J. Furthmüller, Efficiency of ab-initio total energy calculations for metals and semiconductors using a plane-wave basis set. *Comput. Mater. Sci.* **6**, 15–50 (1996).

3.	G. Kresse, J. Hafner, Ab initio molecular dynamics for liquid metals. *Phys. Rev. B* **47**, 558 (1993).

4.	G. Kresse, J. Furthmüller, Efficient iterative schemes for ab initio total-energy calculations using a plane-wave basis set. *Phys. Rev. B* **54**, 11169 (1996).

5.	P. E. Blöchl, Projector augmented-wave method. *Phys. Rev. B* **50**, 17953 (1994).

6.	G. Kresse, D. Joubert, From ultrasoft pseudopotentials to the projector augmented-wave method. *Phys. Rev. B* **59**, 1758 (1999).

7.	J. P. Perdew, K. Burke, M. Ernzerhof, Generalized Gradient Approximation Made Simple. *Phys. Rev. Lett.* **77**, 3865 (1996).

8.	G. Henkelman, ; Blas, P. Uberuaga, H. Jónsson, B. P. Uberuaga, H. Jó, A climbing image nudged elastic band method for finding saddle points and minimum energy paths. *J. Chem. Phys.* **113**, 9901–9904 (2000).

9.	A. Aatiq, M. Ménétrier, L. Croguennec, E. Suard, C. Delmas, On the structure of Li 3 Ti 2 (PO 4 ) 3. *J. Mater. Chem.* **12**, 2971–2978 (2002).

10.	F. Yin, Z. Zhang, Y. Fang, C. Sun, Insight into the mechanism of Li ion diffusion in fluorine-doped Li1.3Al0.3Ti1.7(PO4)3 as an electrolyte for solid lithium metal batteries. *J. Energy Storage* **73**, 108950 (2023).

11.	M. Kim, S. Kim, J. Kim, Integrated NF-MD and CCU system for sustainable lithium recovery from brine: Techno-economic and life cycle assessment. *Chemical Engineering Journal* **522**, 167508 (2025).

12.	D. Ankoliya, A. Mudgal, M. K. Sinha, V. Patel, J. Patel, Techno-economic analysis of a hybrid electrodialysis–batch reverse osmosis process for brackish water desalination. *AQUA - Water Infrastructure, Ecosystems and Society* **72**, 593–607 (2023).
